# Blood Flow Restriction in Upper‐Extremity Rehabilitation: A Systematic Review of Clinical Studies

**DOI:** 10.1002/ars2.70130

**Published:** 2026-10-26

**Authors:** Mitchell J. Christiansen, Sundas Shaikh, Mary K. Mulcahey

**Affiliations:** ^1^ Department of Orthopaedic Surgery and Rehabilitation Loyola University Medical Center Maywood Illinois U.S.A.; ^2^ Loyola Stritch School of Medicine Maywood Illinois U.S.A.

## Abstract

**Purpose:**

To evaluate the effects of blood flow restriction (BFR) therapy on upper‐extremity strength, pain, range of motion, and functional outcomes.

**Methods:**

This systematic review followed the 2009 Preferred Reporting Items for Systematic Reviews and Meta‐Analyses guidelines. PubMed, Embase, and Scopus were searched from January 2000 through August 2025 for studies evaluating upper‐extremity BFR during resistance or therapeutic exercise. Studies were included if they were randomized controlled trials or prospective studies published in English that reported pre‐ and postintervention outcomes of strength, pain, range of motion, muscle morphology, or validated functional scores.

**Results:**

A total of 492 studies were identified, 5 of which met inclusion criteria, encompassing 128 participants. Four studies (80%) were randomized controlled trials, and 1 study (20%) was a prospective cohort study. Clinical indications for the use of BFR included rotator cuff tendinopathy, lateral epicondylitis, distal radius fracture rehabilitation, and postoperative shoulder rehabilitation. Four of 5 studies (80%) showed statistically significant BFR‐related improvements in strength, with the remaining study reporting within‐group improvements that did not reach between‐group significance. Three of 4 studies (75%) that assessed pain reported significant reductions favoring BFR, with the fourth showing clinically within‐group pain reductions in both BFR and control. Notable findings included a significant between‐group improvement in internal rotation strength at 60°/s favoring BFR in rotator cuff tendinopathy (*P* = .04) and significant pain reduction in distal radius fracture rehabilitation (visual analog scale: 5.5 → 1.5, *P* = .03).

**Conclusions:**

In this systematic review, 4 of 5 studies (80%) reported statistically significant strength improvements favoring BFR, and 3 of 4 studies (75%) assessing pain showed significant reductions favoring BFR, with no serious adverse events reported. These findings support BFR as a promising adjunct in upper‐extremity rehabilitation; however, heterogeneity in study design and intervention parameters limits definitive conclusions.

**Level of Evidence:**

Level IV, systematic review of Level I and IV studies.

## Graphical abstract

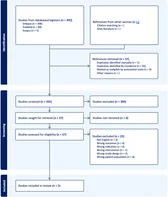

Blood flow restriction (BFR) therapy is a rehabilitation technique that involves vascular occlusion combined with resistance training or exercise. The use of this rehabilitation modality has increased substantially in recent years. The physiological adaptations are thought to result from synergistic effects because metabolic stress induced by vascular occlusion and mechanical tension generated during resistance training together promote muscle hypertrophy and strength.^1^, ^2^, ^3^, ^4^, ^5^, ^6^ In particular, the ischemic and hypoxic environment created by BFR amplifies the accumulation of metabolites, which serve as key mediators of muscular hypertrophy. Most previous studies related to BFR have centered on lower‐extremity applications, mainly focusing on athletes and patients recovering from knee surgery or anterior cruciate ligament reconstruction.^7^, ^8^, ^9^ Conversely, the utilization of BFR therapy as part of upper‐extremity rehabilitation has not been comprehensively studied.

In 2022, Wortman et al. highlighted the developing interest in the use of BFR as a supplement to standard therapy in upper‐extremity injuries.^10^ The authors identified benefits in muscle strength, pain reduction, and improved patient‐reported outcomes. However, the review was limited by the paucity of randomized controlled trials (RCTs) at that time; the inclusion of heterogeneous study designs, such as case series and feasibility studies; and the variability in outcome reporting.^10^ Another systematic review by Tollefson et al. in 2025 reviewed Level I and II studies in healthy athletes and found that lower‐extremity BFR significantly improved strength and muscle size, often yielding results comparable to high‐load resistance training.^8^ Despite these promising findings, few studies have focused on upper‐extremity applications of BFR, and the existing literature remains heterogeneous and underpowered to guide clinical practice.

Recently, a scoping review by Sinnott et al. examined 17 clinical trials on the use of BFR for upper‐extremity injuries.^11^ Patients across the included studies ranged from healthy adults and athletes to rehabilitation populations, including individuals with rotator cuff tendinopathy and postoperative upper‐extremity conditions, as well as sport‐specific cohorts such as climbers and collegiate pitchers. The authors found that BFR not only increased muscle hypertrophy and strength but also yielded adaptations in muscles that were proximal or contralateral to the location of the occlusive device.^11^

The purpose of this study was to evaluate the effects of BFR therapy on upper‐extremity strength, pain, range of motion, and functional outcomes. We hypothesized that BFR supplementation would produce improvements in upper‐extremity strength, pain, and validated functional outcomes.

## METHODS

The systematic review was conducted in compliance with the 2009 Preferred Reporting Items for Systematic Reviews and Meta‐Analyses guidelines. The eligibility criteria and the review question were defined before the review was conducted. We sought studies evaluating the effects of BFR applied to the upper extremity during resistance exercise or therapeutic exercise and reporting pre‐ to postintervention clinical outcomes.

Inclusion criteria were as follows: RCTs or prospective cohort studies; participants who were healthy adults, athletes, or patients undergoing upper‐extremity rehabilitation for any upper‐extremity injury or pathology, managed either operatively or nonoperatively; use of a pneumatic cuff or tourniquet applying BFR to the upper extremity during resistance or therapeutic exercise; interventions lasting at least 4 weeks with a defined training frequency and occlusion pressure; reporting of pre‐ and postintervention outcomes related to strength, pain, range of motion, tendon morphology, or validated functional scores; and full‐text human studies published in English between January 2000 and August 2025.

Exclusion criteria were as follows: case reports, case series, conference abstracts, narrative reviews, technical notes, feasibility studies, or animal or cadaveric studies; studies evaluating lower‐extremity or whole‐body training without upper‐extremity‐specific outcomes; interventions that did not use BFR or used devices without calibrated occlusion; patient populations with conditions unrelated to upper‐extremity rehabilitation; studies lacking pre‐ to postintervention clinical outcomes; and articles not written in English or without accessible full text.

### Eligible Studies

Eligible studies enrolled healthy participants or patients undergoing upper‐extremity rehabilitation without age restriction, used a pneumatic cuff or tourniquet applied before or during exercise, and released afterward. The following databases were searched: PubMed, Embase, and Scopus. Searches spanned from January 2000 through August 2025, which preceded data extraction. The search strategy used was the following: ((blood flow restriction) OR (BFR) OR (occlusion training) OR (vascular occlusion) OR (restricted blood flow)) AND ((upper extremity) OR (shoulder) OR (elbow) OR (wrist) OR (hand) OR (rotator cuff) OR (tendinopathy) OR (epicondylitis) OR (fracture) OR (labrum) OR (Bankart)) AND ((rehabilitation) OR (physical therapy) OR (therapeutic exercise) OR (resistance training) OR (postoperative) OR (recovery)) AND ((randomized) OR (prospective) OR (controlled trial)).

### Study Selection

All the studies were screened in Covidence by 2 reviewers (M.C., S.S.) independently, including the titles, abstracts, and full‐text articles. Any discrepancies in the reviews were discussed among the reviewers, and unresolved issues were addressed by the senior author (M.M.). The full reference list for all included studies was manually screened to identify any additional studies that met inclusion criteria.

### Risk‐of‐Bias Assessment

Methodological quality was assessed for all included studies using the Methodological Index for Non‐Randomized Studies criteria.^12^ Each study was evaluated across 12 items, with each item scored 0 (not reported), 1 (reported but inadequate), or 2 (reported and adequate), yielding a maximum score of 24. Scores below 14 were considered poor quality, 15 to 20 moderate quality, and 21 to 24 high quality.

### Data Analysis

In the included studies, BFR was applied as an adjunct to structured rehabilitation programs across a range of upper‐extremity conditions, including rotator cuff tendinopathy, lateral elbow tendinopathy, distal radius fracture rehabilitation, and postoperative shoulder stabilization. Outcome measures were tailored to each clinical population and encompassed upper‐extremity strength, tendon morphology, pain, wrist and shoulder range of motion, and condition‐specific functional indices including the Disabilities of the Arm, Shoulder, and Hand, Patient‐Rated Wrist Evaluation (PRWE), Patient‐Rated Tennis Elbow Evaluation (PRTEE), and QuickDASH.

Given the clinical and methodological heterogeneity across included studies, quantitative pooling of results was not feasible. Data were extracted independently by 2 reviewers and organized in tabular form in Excel (Microsoft, Redmond, WA). Results are presented descriptively, with between‐group and within‐group findings reported as extracted from each primary source.

## RESULTS

A total of 492 studies were identified through the database search, with 398 from Embase, 89 from PubMed, and 5 from Scopus. Of these records, 57 were duplicates and were removed from the search. An additional 399 studies were removed because they did not meet the inclusion criteria. The full text for 27 studies was reviewed, of which 5 studies met the inclusion criteria and were included in the final analysis (Figure 1). The most common reasons for full‐text exclusion were the absence of pre‐ and postintervention outcome data, study designs not meeting inclusion criteria (case reports, narrative reviews, or feasibility studies), and incorrect patient populations (studies with healthy participants only).

**FIGURE 1:**
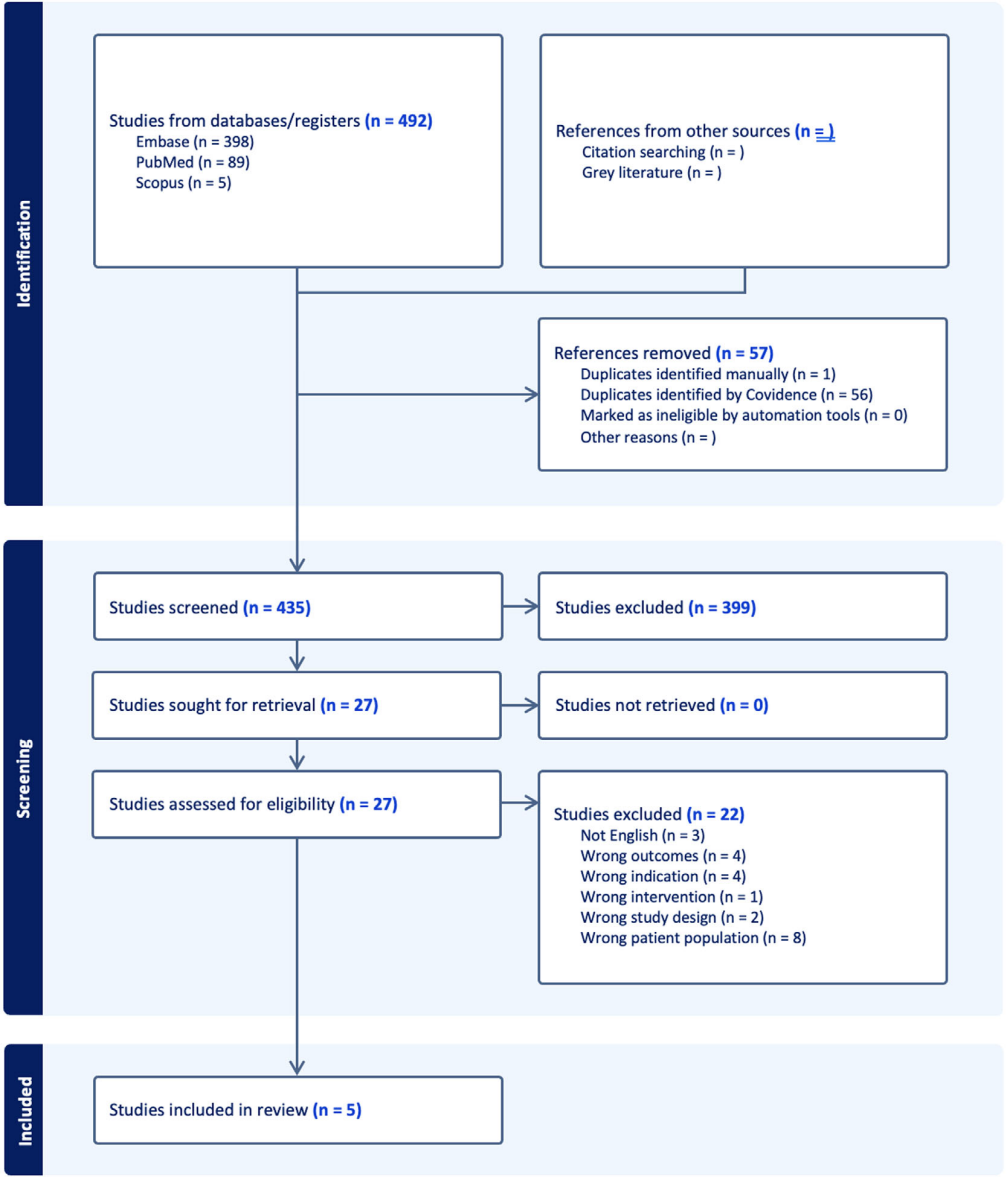
Preferred Reporting Items for Systematic Reviews and Meta‐Analyses flow diagram showing the systematic review of the literature for studies investigating the use of blood flow restriction for upper‐extremity injuries.

### Study Characteristics

Across the 5 studies, a total of 128 patients were included based on extracted reports, with sample sizes from 13 to 46, with 71 men and 57 women of the studies that reported sex. None of the included studies reported outcomes stratified by sex; therefore, a sex‐disaggregated analysis was not performed. Four studies (80%) were RCTs, and 1 study (20%) was a prospective cohort study. Follow‐up windows generally spanned from 6 to 12 weeks, with some extending to later time points (Table 1).

**TABLE 1:** Characteristics of Included Studies Investigating BFR Therapy in Upper‐Extremity Rehabilitation

**Author, Year**	**Level of Evidence**	**Country**	**Population/Indication**	**Men/Women, N**	**Follow‐Up**	**Cuff Site and Width**	**Occlusion Pressure**	**Load (%1‐RM)**	**Sets × Reps**	**Frequency**	**Duration**
Kara (2024)^13^	I (RCT)	Turkey	Rotator cuff tendinopathy	7/7 (14)	8 wk	Proximal arm, 5 cm‐wide pneumatic cuff	~50% LOP	20%‐30%	4 × (30/15/15/15)	2×/wk	8 wk
Fan (2023)^14^	I (RCT)	China	Operative distal radius fracture rehab	17/18 (35)	12 wk	Proximal arm, 5 cm cuff	40%‐80% AOP (~120 mmHg)	20%	4 × (30/15/15/15)	5×/wk	4 wk
Karanasios (2022)^16^	I (RCT, sham‐controlled)	Greece/United Kingdom	Lateral elbow tendinopathy	24/22 (46)	6 wk, 12 wk	Arm cuff	40%‐50% LOP	30%	4 × (30/15/15/15)	2×/wk	6 wk
Cancio (2019)^15^	I (RCT)	United States	Nonoperative distal radius fracture rehab	6/7 (13)	8 wk	Arm cuff	50% LOP	20%	4 × (30/15/15/15)	3×/wk	8 wk
McGinniss (2022)^17^	IV (case series)	United States	Postop shoulder rehab	17/3 (20)	12 wk, 6 mo	Arm cuff (18 × 4.5 inch)	50% LOP	30%	4 × (30/15/15/15)	3×/wk	6 wk

*Note*: Details include study design, population, sample size, follow‐up duration, cuff parameters, occlusion pressure, exercise intensity, frequency, and total program length.

1‐RM, 1‐repetition maximum; AOP, arterial occlusion pressure; BFR, blood flow restriction; LOP, limb occlusion pressure; mo, months; RCT, randomized controlled trial; wk, weeks.

Across the 5 included studies, patient populations spanned a variety of clinical contexts. One study evaluated patients with rotator cuff tendinopathy.^13^ Two studies examined patients undergoing physical therapy after distal radius fractures; of these, 1 was operative, and 1 was nonoperative.^14^, ^15^ A fourth study assessed patients with lateral elbow tendinopathy using a sham‐controlled design.^16^ The final study followed patients recovering from shoulder procedures, integrating BFR into structured rehabilitation protocols.^17^

### Intervention Protocols

The intervention protocols varied across the 5 included studies. Kara et al. applied BFR therapy to the proximal arm in patients with rotator cuff tendinopathy using a 5 cm cuff inflated to 50% of limb occlusion pressure (LOP), combined with low‐load resistance training twice weekly for 8 weeks.^13^ Fan et al. investigated BFR as a component of postoperative rehabilitation after open reduction internal fixation of distal radius fractures, applying a 5 cm cuff to the proximal arm at 40% to 80% arterial occlusion pressure (AOP) (~120 mmHg), with training performed 5 times per week for 4 weeks.^14^ Karanasios et al. studied patients with lateral elbow tendinopathy, using a proximal arm cuff inflated to 40% to 50% LOP during low‐load resistance exercises 2 times per week for 6 weeks.^16^ Cancio et al. evaluated BFR therapy during nonoperative distal radius fracture rehabilitation, applying 50% LOP with low‐load resistance exercises 3 times weekly for 8 weeks.^15^ McGinniss et al. investigated postoperative shoulder stabilization surgery rehabilitation with an 18 × 4.5 cm cuff inflated to 50% LOP, performing 4 sets of resistance exercises (30/15/15/15 repetitions) 3 times weekly for 6 weeks.^17^ Procedures included arthroscopic labral and capsuloligamentous stabilization, most frequently Bankart repair, with no concomitant rotator cuff repairs. BFR therapy was initiated at the outset of rehabilitation across all studies, with the exception of Fan et al., who delayed initiation by 3 to 7 days postoperatively, and Cancio et al., in whom BFR began at 6 weeks postinjury after cast removal and transition to a wrist splint.^14^, ^15^

Occlusion pressure was individually calibrated in 4 of the 5 included studies. Kara et al., Karanasios et al., Cancio et al., and McGinniss et al. each determined LOP per participant using Doppler ultrasound or an automated tourniquet system, with cuff pressure set as a percentage of each individual's measured value.^13^, ^15^, ^16^, ^17^ Fan et al. applied a fixed pressure of 120 mmHg across all participants, described as approximating 40% to 80% AOP, without individualized calibration.^14^ LOP represents the minimum pressure required to fully occlude arterial flow at rest; AOP represents the pressure at which complete arterial occlusion is achieved.

### Outcomes

The outcomes assessed were heterogeneous and tailored to the clinical population in each study. Kara et al. measured isokinetic shoulder strength at 60 and 180°/s as a ratio of the subjects body weight, supraspinatus and biceps muscle thickness via ultrasound, and pain using a visual analog scale.^13^ Fan et al. reported wrist range of motion, wrist strength, grip strength, and pain scores.^14^ Karanasios et al. evaluated changes in pain‐free grip strength, Patient‐Rated Tennis Elbow Evaluation, and pain reduction among patients with lateral elbow tendinopathy.^16^ Cancio et al. assessed pain scores, grip strength, pinch strength, the PRWE, and the Disabilities of the Arm, Shoulder, and Hand.^15^ McGinniss et al. reported shoulder strength as a ratio of body weight compared with the contralateral limb, range of motion, and functional recovery using the QuickDASH (Table 2).^17^

**TABLE 2:** Clinical Outcomes of Included Studies Evaluating BFR Therapy in the Upper Extremity

**Author, Year**	**Clinical Outcomes**	**Control Group Results (Pre → Post) (95% CI)**	**BFR Group Results (Pre → Post) (95% CI)**	**Significant Findings**
Kara (2024)^13^	strength 60°/s, Nm/kg	0.31 (0.25‐0.37) → 0.34 (0.27‐0.42)	0.32 (0.28‐0.37) → 0.37 (0.32‐0.43)	NS
	IR strength 60°/s, Nm/kg	0.42 (0.30‐0.54) → 0.43 (0.32‐0.54)	0.44 (0.38‐0.57) → 0.50 (0.43‐0.57)	**Significant increase with BFR (** ***P*** ** < .05)**
	ER strength 180°/s, Nm/kg	0.26 (0.21‐0.31) → 0.32 (0.25‐0.39)	0.28 (0.24‐0.32) → 0.32 (0.26‐0.37)	NS
	IR strength 180°/s, Nm/kg	0.39 (0.28‐0.50) → 0.42 (0.31‐0.53)	0.39 (0.34‐0.43) → 0.45 (0.39‐0.52)	NS
	Biceps brachii muscle thickness, mm	2.72 (2.37‐3.06) → 2.62 (2.24‐2.99)	2.64 (2.36‐2.91)→ 2.87 (2.51‐3.22)	**Significant increase with BFR (** ***P*** ** < .05)**
	Supraspinatus muscle thickness, mm	1.97 (1.64‐2.30) → 2.10 (1.87‐2.32)	2.11 (1.92‐2.31) → 2.35 (2.12‐2.58)	NS
	Pain (VAS)	5.41 (4.44‐6.39) → 1.12 (0.46‐1.78)	5.00 (3.85‐6.15) → 0.76 (0.26‐1.25)	**Both control and BFR showed reduction in pain (** ***P*** ** < .05)**
Fan (2023)^14^	Wrist ROM, °	52.39 (45.56‐58.55) → 38.33 (32.95‐42.76) → 14.89 (11.90‐17.85)	53.53 (47.73‐58.88) → 31.41 (26.17‐36.29) → 14.12 (11.15‐16.82)	NS
	Grip strength (% of healthy side)	0.21 (0.16‐0.2) → 0.35 (0.30‐0.40) → 0.62(0.58‐0.65)	0.19 (0.16‐0.2) → 0.38 (0.33‐0.42) → 0.62 (0.59‐0.65)	NS
	Wrist extension strength (% of healthy side)	0.25 (0.21‐0.30) → 0.34 (0.30‐0.38) → 0.52 (0.50‐0.55)	0.26 (0.22‐0.29)→ 0.49 (0.44‐0.55) → 0.72 (0.68‐0.76)	**Strength improved at 4 wk (** ***P*** ** < .05)**
	Wrist flexion strength (% of healthy side)	0.28 (0.25‐0.32) → 0.38 (0.34‐0.42) → 0.52 (0.48‐0.56)	0.30 → (0.25‐0.35) 0.50 → (0.45,0.55) 0.70 → (0.67‐0.73)	**Strength improved at 4 wk (** ***P*** ** < .05)**
	Pain (VAS, 4 wk)	1.28 (0.94‐1.59)	0.06 (<0.01‐0.19)	**Pain improved at 4 wk (** ***P*** ** < .05)**
Karanasios (2022)^16^	Pain‐free grip strength (isometric torque, Nm/kg)	0.75 (0.65‐0.85) → 0.73 (0.63‐0.83) → 0.80 (0.70‐0.91)	0.71 (0.61‐0.81) → 0.93 (0.83‐1.03) → 0.94 (0.84‐10.4)	**Strength improved with BFR (** ***P*** ** < .05)**
	Pain (VAS)	6.64 (5.71‐7.5) → 4.62 (3.65‐5.59) → 3.27 (2.3‐4.24)	7.47 (6.5‐8.4) → 3.47 (2.52‐4.42) → 1.73 (0.78‐2.68)	**Greater reduction with BFR at 12 wk (** ***P*** ** < .05)**
	PRTEE	36.99 (31.29‐41.87) → 24.68 (18.74‐30.61) → 20.24 (14.31‐26.18)	38.74 (33.13‐44.55) → 12.75 (6.95‐18.5) → 5.01 (0.79‐10.81)	**Greater reduction in score with BFR (** ***P*** ** < .05)**
Cancio (2019)^15^	Pain (VAS)	4.4 → 2.0 → 2.1	5.5 → 3.7 → 1.5	**Greater reduction in pain with BFR (** ***P*** ** < .05)**
	PRWE	47.8 → 30.2 → 17.0	66.8 → 31.1 → 8.9	**Greater reduction with BFR (** ***P*** ** < .05)**
	DASH score	44.1 → 25.5 → 20.1	51.3 → 26.7 → 8.8	NS
	Grip strength	26.9 → 42.9 → 55.1	24.2 → 39 → 56.2	NS
	Pinch strength	7.6 → 10.4 → 12.3	7.3 → 12.5 → 14.3	NS
McGinniss (2022)^17^	ER strength (kg/body mass in kg)	No control	Within‐group difference: 0.049 (0.021‐0.077)	**Significant improvement with BFR (** ***P*** ** < .05)**
	Abduction strength (kg/body mass in kg)		Within‐group difference: 0.079 (0.050‐0.108)	**Significant improvement with BFR (** ***P*** ** < .05)**
	IR strength (kg/body mass in kg)		Within‐group difference: 0.060 (0.028‐0.093)	**Significant improvement with BFR (** ***P*** ** < .05)**
	ER ROM, °		Within‐group difference: 24.56 (13.20‐35.91)	**Significant improvement with BFR (** ***P*** ** < .05)**
	Flexion ROM, °		Within‐group difference: 20.94 (14.00‐27.90)	**Significant improvement with BFR (** ***P*** ** < .05)**
	IR ROM, °		Within‐group difference: 15.11 (6.04‐24.18)	**Significant improvement with BFR (** ***P*** ** < .05)**
	SANE		Within‐group difference: 17.7 (9.4‐25.9)	**Significant improvement with BFR (** ***P*** ** < .05)**
	SPADI		Within‐group difference: −31.1 (−44.2 to −18.0)	**Significant improvement with BFR (** ***P*** ** < .05)**
	QuickDASH		Within‐group difference: −6.9 (−9.2 to −4.6)	**Significant improvement with BFR (** ***P*** ** < .05)**

*Note*: Pre‐ and postintervention results are shown for both control and BFR groups, including measures of strength, pain, ROM, muscle thickness, and validated functional outcomes. Significant findings are indicated when reported by the original authors. Bolded elements have *P* < .05.

BFR, blood flow restriction; CI, confidence interval; DASH, Disabilities of the Arm, Shoulder, and Hand; ER, external rotation; IR, internal rotation; NS, not significant; PRTEE, Patient‐Rated Tennis Elbow Evaluation; PRWE, Patient‐Rated Wrist Evaluation; ROM, range of motion; SANE, Single Assessment Numeric Evaluation; SPADI, Shoulder Pain and Disability Index; VAS, visual analog scale; wk, weeks.

### Risk‐of‐Bias Assessment

Although all studies were described as randomized or prospective in design, limitations were common across the included literature: blinding of treating clinicians was generally not feasible given the nature of the intervention, and randomization procedures were inconsistently described. The mean Methodological Index for Non‐Randomized Studies score across included studies was 20. Based on the Methodological Index for Non‐Randomized Studies scoring, our included studies were of moderate quality, with several meeting key methodological criteria but lacking consistency in areas such as consecutive patient inclusion and control group adequacy (Table 3).

**TABLE 3:** MINORS Criteria

	**First Author, Year**				
	**Cancio, 2019** ^16^	**Fan, 2023** ^14^	**Kara, 2024** ^13^	**Karanasios, 2022** ^15^	**McGinniss, 2022** ^17^
A clearly stated aim	2	2	2	2	2
Inclusion of consecutive patients	2	1	1	1	1
Prospective collection of data	2	2	2	2	2
Endpoints appropriate to the aim of the study	2	2	2	2	2
Unbiased assessment of the study endpoint	1	2	2	2	0
Follow‐up period appropriate to the aim of the study	2	2	2	2	2
Loss to follow‐up less than 5%	2	1	2	1	1
Prospective calculation of the study size	0	2	2	2	0
An adequate control group	2	2	2	2	0
Contemporary group	2	2	2	2	0
Baseline equivalence of groups	2	2	2	2	0
Adequate statistical analyses	2	2	2	2	2
Results	21/24	22/24	23/24	22/24	12/24

MINORS, Methodological Index for Non‐Randomized Studies.

### Adverse Events

No serious adverse events attributable to BFR were reported across the included studies. McGinniss et al. explicitly stated that no adverse events occurred.^17^ Kara et al. reported no adverse events.^13^ Cancio et al. reported no complications, with all patients tolerating the program and returning to preinjury occupational function.^15^ Fan et al. reported that all participants completed the trial without complications, although minor discomfort during sessions was noted.^14^ Karanasios et al. formally monitored for delayed onset muscle soreness, excessive pain, numbness, bruising, and tingling; no adverse events were reported, and no dropouts were attributable to side effects.^16^ No patients across any study required escalation to operative management.

## DISCUSSION

The most important finding of this study was that BFR therapy produced significant reductions in pain in patients with distal radius fractures and lateral elbow tendinopathy; improved functional outcomes, including PRWE, Disabilities of the Arm, Shoulder, and Hand, and QuickDASH, in distal radius fracture and postoperative shoulder rehabilitation; and showed significant strength improvements in 4 of 5 included studies. In patients with rotator cuff tendinopathy, BFR produced significant between‐group improvements in biceps brachii muscle thickness and internal rotation strength at 60°/s, suggesting that proximal occlusion can elicit hypertrophic and strength adaptations even in the muscles located above the cuff. In lateral elbow tendinopathy, BFR training resulted in greater pain reduction than sham BFR, with significant between‐group improvement in pain‐free grip strength and Patient‐Rated Tennis Elbow Evaluation scores at 6 weeks, supporting its use over low‐load exercise alone.

Two studies investigating rehabilitation after distal radius fractures reported consistent directional benefits. Fan et al. and Cancio et al. both observed improvements in pain, wrist range of motion, and disability scores in both groups, although BFR produced greater functional gains, most notably in wrist flexion and extension strength at 4 weeks and PRWE scores at final follow‐up.^14^, ^15^ These findings are particularly relevant given the constraints of early postfracture rehabilitation, where high‐load training is not feasible. In postoperative Bankart repair shoulder rehabilitation, BFR produced significant within‐group improvements in strength, range of motion, and QuickDASH scores.^17^ Taken together, these findings suggest that BFR may serve as a useful adjunct across a range of upper‐extremity conditions; however, the limited number of studies and heterogeneity in populations and protocols preclude definitive conclusions.

Considerable heterogeneity was present among the included studies, reflecting differences in study design, populations, and intervention parameters. Fan et al., the largest study by sample size, implemented a high‐frequency, short‐duration program with broad occlusion pressures (40%‐80% AOP), which may have attenuated between‐group differences.^14^ Cancio et al. differed as a small pilot study with limited statistical power, although its findings were directionally consistent with larger trials.^15^ In contrast, Kara et al. and McGinniss et al. used structured rehabilitation protocols with defined follow‐up and reported clearer improvements in pain and function.^13^, ^17^ Variability in cuff width, occlusion calibration, and exercise dosing further contributed to outcome differences across studies, underscoring the need for standardized methodology in future investigations of upper‐extremity BFR therapy. No serious adverse events were reported across any included study, and no patients required escalation to operative management, supporting BFR as a safe adjunct to upper‐extremity rehabilitation across a range of clinical populations and pathologies.

Several prior reviews have examined the effects of BFR training in healthy or athletic populations, largely outside of clinical rehabilitation contexts.^10^, ^18^, ^19^, ^20^, ^21^, ^22^ In a 2016 systematic review, Scott et al. showed that combining BFR with traditional resistance training enhances strength, muscle hypertrophy, and athletic performance in healthy athletes, with most studies focusing on lower‐extremity or whole‐body training rather than injury recovery.^18^ Similarly, in a 2017 review by Scott et al., analyses of BFR use in athletes confirmed that low‐load BFR can produce comparable strength gains to high‐load training while minimizing joint stress, supporting its safety and efficacy for performance enhancement.^20^ More recent upper‐extremity‐specific reviews by Jing et al. and Pavlou et al. have expanded these findings, showing that low‐load BFR training can improve muscle strength and hypertrophy in the arms while reducing the mechanical load required to achieve these adaptations.^23^, ^24^

However, most of these prior studies were conducted in healthy cohorts or athletic settings, with few directly addressing clinical or postoperative upper‐extremity populations. The scoping review by Sinnott et al. broadly summarized the potential applications of BFR in rehabilitation but did not limit inclusion to RCTs or report granular outcomes.^11^ In contrast, our review uniquely focuses on randomized and prospective studies investigating BFR in patients undergoing upper‐extremity rehabilitation, including conditions such as rotator cuff tendinopathy, distal radius fracture recovery, and postoperative shoulder rehabilitation. Despite differing populations, similar conclusions can be drawn, namely, that BFR can reproduce many of the physiological and functional benefits of high‐load training at substantially lower intensities. By isolating higher‐quality clinical evidence, our findings bridge the gap between performance‐based research and rehabilitation‐based application, reinforcing the role of BFR as a promising adjunct in upper‐extremity recovery. Further high‐quality randomized studies are needed to determine the optimal parameters and long‐term benefits of BFR in upper‐extremity rehabilitation.

### Limitations

There are several limitations to this study. The number of eligible studies investigating BFR in upper‐extremity rehabilitation remains small, limiting the strength of evidence and the ability to perform quantitative synthesis. Substantial heterogeneity was present among studies in terms of cuff width, occlusion pressure calibration, exercise type, frequency, and progression criteria, which restricts direct comparison and precludes the identification of an optimal protocol. There is substantial heterogeneity in the pathology and outcomes measured in the participants in the studies included, which limits the condition‐specific pooling of any data, including meta‐analysis. Most included studies had relatively short follow‐up durations, typically 6 to 8 weeks, making it difficult to assess long‐term adaptations or durability of functional gains. Sample sizes in several studies were small and underpowered to detect modest treatment effects. None of the included studies reported sex‐disaggregated outcome data, precluding any analysis of the influence of sex on BFR treatment effects in accordance with Sex and Gender Equity in Research guidelines. Inclusion was restricted to English‐language publications, introducing the possibility of language bias and potential exclusion of relevant studies published in other languages. A formal assessment of overall evidence certainty using the grading of recommendations assessment framework was not performed, which limits the ability to make explicit recommendations regarding the strength of the available evidence. Additionally, although 4 of the 5 studies were RCTs, the overall methodological quality varied, and few trials reported blinding or standardized outcome assessment. Publication bias cannot be excluded because negative or inconclusive findings may be underrepresented in the literature. Finally, this study was not prospectively registered with prospective register of systematic reviews or another registry. This reduces transparency, making it harder to verify whether the review was conducted according to a prespecified plan.

## CONCLUSIONS

In this systematic review, 4 of 5 studies (80%) reported statistically significant strength improvements favoring BFR, and 3 of 4 studies (75%) assessing pain showed significant reductions favoring BFR, with no serious adverse events reported. These findings support BFR as a promising adjunct in upper‐extremity rehabilitation; however, heterogeneity in study design and intervention parameters limits definitive conclusions.

## DISCLOSURES

The author (M.K.M.) declares the following financial interests/personal relationships, which may be considered potential competing interests: M.K.M. receives consulting fees from Arthrex and reports society, committee, or advocacy group, paid or unpaid, from AANA (member of Education Committee and Fellowship Committee), AOSSM (member of Education Committee), ABJS (Chair of Membership Committee), ISAKOS (member of Shoulder Committee, Membership Committee, Chair of Gender and Diversity Task Force, type: Board of Directors or committee member), and JBJS, AJSM, Arthroscopy Journal, and AAOS OrthoInfo (type: Editorial or governing board). The other authors (M.J.C., S.S.) declare that they have no known competing financial interests or personal relationships that could have appeared to influence the work reported in this paper.
